# Whole-genome transcriptome and DNA methylation dynamics of pre-implantation embryos reveal progression of embryonic genome activation in buffaloes

**DOI:** 10.1186/s40104-023-00894-5

**Published:** 2023-07-11

**Authors:** Penghui Fu, Du Zhang, Chunyan Yang, Xiang Yuan, Xier Luo, Haiying Zheng, Yanfei Deng, Qingyou Liu, Kuiqing Cui, Fei Gao, Deshun Shi

**Affiliations:** 1 State Key Laboratory for Conservation and Utilization of Subtropical Agro-Bioresources & Guangxi Key Laboratory of Animal Breeding and Disease Control, Guangxi University, Nanning, 530004 China; 2 College of Animal Science and Technology, Southwest University, Chongqing, 402460 China; 3 Genome Analysis Laboratory of the Ministry of Agriculture, Agricultural Genomics Institute at Shenzhen, Chinese Academy of Agricultural Sciences, Shenzhen, 518120 China; 4 Guangxi Key Laboratory of Buffalo Genetics, Reproduction and Breeding, Guangxi Buffalo Research Institute, Chinese Academy of Agricultural Science, Nanning, 530001 China; 5 Guangxi Academy of Medical Sciences and the People’s Hospital of Guangxi Zhuang Autonomous Region, Nanning, 530016 China; 6 Guangdong Provincial Key Laboratory of Animal Molecular Design and Precise Breeding School of Life Science and Engineering, Foshan University, Foshan, 528225 China; 7 Comparative Pediatrics and Nutrition, Department of Veterinary and Animal Sciences, Faculty of Health and Medical Sciences, University of Copenhagen, DK 1870 C Frederiksberg, Denmark

**Keywords:** Buffalo, DNA methylome, Embryonic genome activation, Maternal-to-zygote transition, Transcriptome

## Abstract

**Background:**

During mammalian pre-implantation embryonic development (PED), the process of maternal-to-zygote transition (MZT) is well orchestrated by epigenetic modification and gene sequential expression, and it is related to the embryonic genome activation (EGA). During MZT, the embryos are sensitive to the environment and easy to arrest at this stage in vitro. However, the timing and regulation mechanism of EGA in buffaloes remain obscure.

**Results:**

Buffalo pre-implantation embryos were subjected to trace cell based RNA-seq and whole-genome bisulfite sequencing (WGBS) to draw landscapes of transcription and DNA-methylation. Four typical developmental steps were classified during buffalo PED. Buffalo major EGA was identified at the 16-cell stage by the comprehensive analysis of gene expression and DNA methylation dynamics. By weighted gene co-expression network analysis, stage-specific modules were identified during buffalo maternal-to-zygotic transition, and key signaling pathways and biological process events were further revealed. Programmed and continuous activation of these pathways was necessary for success of buffalo EGA. In addition, the hub gene, *CDK1*, was identified to play a critical role in buffalo EGA.

**Conclusions:**

Our study provides a landscape of transcription and DNA methylation in buffalo PED and reveals deeply the molecular mechanism of the buffalo EGA and genetic programming during buffalo MZT. It will lay a foundation for improving the in vitro development of buffalo embryos.

**Graphical Abstract:**

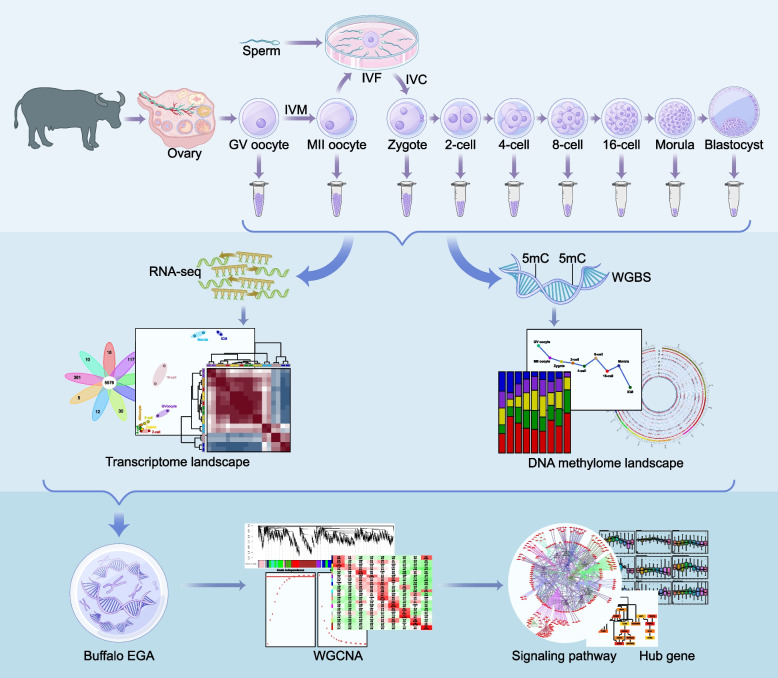

**Supplementary Information:**

The online version contains supplementary material available at 10.1186/s40104-023-00894-5.

## Background

Buffalo (*Bubalus bubalis*) is an important economic animal in tropical and subtropical regions, but the development of the buffalo industry is greatly limited by its low reproductive ability. Embryonic biotechnologies such as in vitro embryo production (IVEP) and somatic cell nuclear transfer (SCNT) can accelerate genetic breeding in buffalo [1] and improve the development of the buffalo industry. However, the low blastocyst development rates (26.5%–39.48%) [2] have compromised its wide-scale application. After fertilization, zygotes will undergo cleavage and their development will transit from the maternal control to the zygotically control, and this process is called the maternal-to-zygotic transition (MZT) and it is related to embryonic genome activation (EGA) [3]. There is increasing evidence that embryos are sensitive to the environment during MZT and are easy to arrest at this stage in vitro [4]. Pre-implantation embryos undergo high dynamics of transcriptional and epigenetic reprogramming during MZT. Several lines of evidence indicate that there are notable interspecific differences in the timing of major EGA. Moreover, although humans and mice share many core transcriptional programs at the pre-implantation embryo development (PED) stage [5–7], the underlying molecular regulatory mechanisms of mammalian PED also are not exactly the same in different species when this was assessed via cross-species transcriptome comparative analysis [8, 9]. Therefore, it is valuable to explore the molecular mechanism of buffalo PED with respect to EGA.

There are few reports concerning PED in buffalo, while the timing of EGA in buffalo are not consistent. Early studies that employed α-amanitin to inhibit RNA polymerase II activity of early buffalo embryos found that most of them were blocked at the 4- to 8-cell stages, suggesting that the timing of buffalo major EGA was at the 8-cell stage [10]. However, Chen et al. [11] applied quantitative proteomics combined with RNA sequencing to reveal the dynamic function of maternally-expressed proteins and genes after parthenogenetic activation of buffalo oocytes, and indicated that EGA may occur between the 8-cell to 16-cell stages. Recent study revealed the transcriptomic profile of in vitro fertilization (IVF) buffalo embryos at four stages (2-cell, 8-cell, morula and blastocyst) via RNA-seq and elucidated the expression patterns of transcription factors in these stages and found that the expression levels of most transcription factor were promoted during the blastocyst stage [12], while provided no information about MZT. Therefore, as these findings have yet identified the regulators of EGA in buffalo embryos, a comprehensive understanding of the complex regulation of the transcriptional and epigenetic events that occur during EGA remains elusive.

In recent years, various low-input sequencing technologies have been used to analyze mammalian PED to fully understand its regulatory mechanisms [13–16]. In this paper, we drew landscapes of transcription and DNA methylation of buffalo pre-implantation embryos by performing trace cell based RNA sequencing and whole-genome bisulfite sequencing (WGBS) analysis of germinal vesicle (GV) oocytes, metaphase II (MII) oocytes as well as seven crucial stages of IVF embryos (zygote, 2-, 4-, 8-, 16-cell, morula and inner cell mass (ICM) from blastocyst). The aim of this study was to assemble a complete gene expression time course spanning buffalo pre-implantation embryogenesis, and to elucidate buffalo EGA and genetic program during buffalo MZT, so as to identify key transcriptional features and epigenic modifications over developmental time and to understand the in-depth molecular mechanisms associated with EGA. This work will lay a foundation for improving the in vitro development of buffalo embryos.

## Methods and materials

### In vitro maturation, fertilization, culture and sample collection

Water buffalo oocytes and embryos were obtained through in vitro maturation (IVM), IVF and in vitro culture (IVC) techniques as previously described [17–19]. In brief, buffalo cumulus-oocyte complexes (COCs) were cultured in droplets of IVM medium for 20–22 h under a humidified 5% CO_2_ in air at 38.5 °C. MII oocytes were then incubated with buffalo frozen-thawed motile spermatozoa (2 × 10^6^ cells/mL) that were selected by the swim-up technique in droplets of in vitro fertilization medium for 22 h under a humidified 5% CO_2_ in air at 38.5 °C. After insemination, zygotes were cultured in droplets of IVC medium with cumulus cell monolayers for 6–7 d under a humidified 5% CO_2_ in air at 38.5 °C. All oocytes and embryos were carefully assessed by using a stereomicroscope. All selected oocytes and embryos were exposed to the 0.5% pronase (10165921001, Roche, Mannheim, Germany) medium for 30–45 s to remove the zona pellucida. The polar bodies of MII oocytes and zygotes were carefully removed by washing, and the ICM was isolated from blastocyst in PBS by mechanical stripping using two sharp needles. The details regarding the collection times of samples at each developmental stage are described in Table S1.

### RNA-seq library construction and sequencing

Full-length RNA-seq libraries for trace cells (hundreds of cells) were prepared using the Smart-seq2 protocol [20] with minor modifications. In brief, total RNA was released from oocytes/embryos using cell lysis buffer (1U RNase Inhibitor and 0.2%Triton-X 100). These were subjected to reverse transcription and template-switching reactions for 10 cycles in Super Script II first-strand buffer with SuperScript II reverse transcriptase (18064014, Invitrogen, Carlsbad, CA, USA). Subsequently, cDNA was amplified for 15 cycles by using KAPA HiFi HotStart ReadyMix (KK2602, KAPA Biosystems, Cape Town, South Africa) and was purified using Ampure XP beads (A63881, Beckman Coulter, Bria, USA). Sequencing libraries were constructed using the TruePrep DNA Library Prep Kit V2 (TD202, Vazyme, Nanjing, China) and then they were quantified using the Agilent Bioanalyzer 2100 (G2947CA, Agilent, Santa Clara, USA) and qPCR. The qualified cDNA libraries were sequenced with 150 bp of paired-end reads by the Illumina HiSeq X Ten (Illumina, San Diego, USA).

### WGBS library construction and sequencing

WGBS libraries for trace cells were prepared according to a previously published protocol for single cell [21] with minor modifications. In brief, oocytes/embryos were lysed in cell lysis buffer (10 mmol/L Tris–HCl pH 7.4 and 2% SDS) for 1 h at 37 °C. EZ DNA Methylation-Gold™ Kit (D5006, Zymo Research, Irvine, USA) was conducted to bisulfite conversion and DNA was eluted by 10 mmol/L Tris–HCl buffer (pH 8.5) (15567–027, Invitrogen, Carlsbad,USA). Then random priming and extension of junction 1 were performed 5 times with Klenow (3′→5′ exo-) in the 1× Blue Buffer (P7010-HC-L, Enzymatics, Beverly, MA, USA). Subsequently, DNA was incubated with exonuclease I (M0293L, NEB, Ipswich, England) for 1 h at 37 °C and purified with Agencourt Ampure XP (A63881, Beckman Coulter, Bria, USA). After capturing with M-280 Streptavidin Dynabeads (11206D, Life Technologies, Carlsbad, USA) and washing with 0.1 mol/L NaOH, the extension products were then used to performe random priming and extension of junction 2 using Klenow (3′→5′ exo-) in the 1× Blue Buffer (P7010-HC-L, Enzymatics, Beverly, USA). Libraries were then amplified by PCR and purified. After the quality was assessed by using the Agilent Bioanalyzer 2100 (G2947CA, Agilent, Santa Clara, USA) and QPCR, the WGBS libraries were sequenced by the Illumina HiSeq X Ten (Illumina, San Diego, USA).

### Transcriptome data analysis

Raw transcriptome reads were cleaned and then mapped to the reference genome with Hisat2 [22]. Reads mapping to the genes were counted by HTSeq-count [23]. Intergroup differential expression analysis was performed by DEseq2 [24]. Differentially expressed genes (DEGs) were identified with a threshold of fold change > 2 and false discovery rate < 0.05. Global expression principal component analysis (PCA) and cluster analysis of all the samples was conducted based on normalized expression levels (FPKM) by R package ade4. In maternal suppress and first expression gene analysis, the genes with FPKM > 5 were identified as expressed genes in the corresponding samples.

### WGBS data analysis

Low-quality reads and sequencing adapters were removed by using cutadapt (version 1.9) [25]. BSMAP (version 2.73) was used to mapping the clean reads to the reference genome [26]. The commonly covered CpG sites with sequencing depths ≥ 5 × in all the samples were selected for further analysis. Methylation level on single base resolution throughout the genome was calculated by “methratio.py” of BSMAP. The metilene (version 0.2–7) software was used to identify the differentially methylated regions (DMRs) between two groups [27] with the following criteria: ≥ 10 CpG sites in the DMR, neighboring CpG sites distance ≤ 300 bp, methylation level difference > 0.1, and *Q*-value < 0.05 using the Benjamini–Hochberg method [28].

### Weighted gene co-expression network analysis (WGCNA)

The R package for WGCNA was employed to construct co-expression network. Firstly, all the expressed genes were sorted by their standard deviations of expression values, and the top 8,000 genes were selected for WGCNA. Based on the undirected network model, the weighted correlation was calculated, and β = 14 (scale-free *R*^2^ = 0.7192) was determined as the ideal soft threshold power to construct the scale-free topology network. Then, the gene modules were identified using an unsupervised hierarchical clustering analysis, and the dynamic tree (dendrogram) was constructed. The module eigengene E represented the gene expression profile of each module.

### ClueGO enrichment analysis for stage-specific modules and identification of the top 20 hub genes

Modules with Pearson’s correlation coefficient (*r*) > 0.4 and *P* < 0.05 were selected as stage-specific modules. Kyoto Encyclopedia of Genes and Genomes (KEGG) pathway enrichment analysis and Gene Ontology (GO) biological process (BP) term enrichment were performed by using ClueGO (version 2.5.9) plugin in Cytoscape software (version 3.9.1). The interaction network among hub genes at each buffalo embryonic developmental stage was created through the online STRING database (database version: 11.5). The top 10 hub genes were ranked by cytoHubba plugin in Cytoscape software by using the Stress method.

### Quantitative real-time PCR

Total RNA was extracted using RNA-easy Isolation Reagent (R701, Vazyme, Nanjing, China) from 30 embryos. The first-strand cDNA was synthesized using HiScript III RT SuperMix for qPCR kit (R323-01, Vazyme, Nanjing, China), and qRT-PCR was performed using ChamQ Universal SYBR qPCR Master Mix kit (Q711, Vazyme, Nanjing, China) on a LightCycler480 (Roche, Penzberg, Germany) by using the following program: 95 °C for 30 s; 40 cycles of 95 °C for 10 s, 60 °C for 30 s, and 95 °C for 15 s, 60 °C for 60 s, 95 °C for 15 s. The primer pairs are listed in Table S2. The gene expression levels were quantified using the 2^-ΔΔCT^ method.

## Results

### Construction of the transcriptome landscape during buffalo PED

**Fig. 1:**
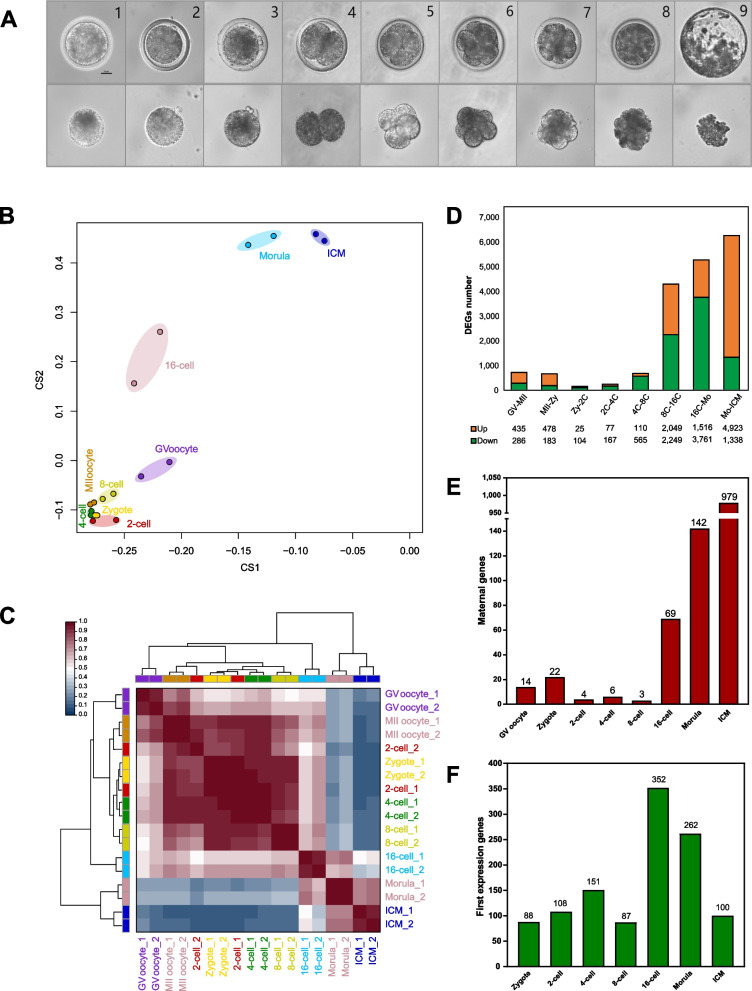
The transcriptome landscape during buffalo PED. **A** Microscopy images of buffalo oocytes and embryos. The top images are the oocytes/embryos with their zona pellucida. The bottom images are the zona-free oocytes/embryos. From 1 to 9: GV oocyte, MII oocyte, Zygote, 2-cell, 4-cell, 8-cell, 16-cell, Morula, blastocyst (in the lower subfigure the ICM was isolated from the blastocyst). **B** Principal component analysis (PCA) of the transcripts for all developmental stages. **C** Unsupervised hierarchal clustering and heatmap of duplicate samples. **D** The numbers of DEGs in consecutive developmental stages during buffalo PED. **E** The number of expression suppression of maternal genes at each embryonic developmental stage. **F** The number of first expression genes at each embryonic developmental stage

To understand the dynamics of transcriptional profiling during buffalo PED, the 18 samples at the 2 stages of buffalo oocytes and 7 stages of pre-implantation embryos were subjected to trace cell based RNA-seq (Fig. 1A). The results showed that approximately 168.9 Gb clean data (an average of 9.08 Gb per sample) were obtained (Table S3) and the average clean reads rate was 85.24%. The total numbers of detectable genes ranged from 10,101 to 14,090 in oocytes and early embryos at different developmental stages, of which 5,576 genes were co-expressed at all stages (Fig. S1).

PCA showed that the gene expression patterns in the MII oocytes and zygotes as well as the 2-, 4- and 8-cell stages were remarkably clustered together. A minor cluster of expression patterns, consist of the morula and ICM, was divergent from the major cluster and the 16-cell stage was the transitional phase in between (Fig. 1B). Similar results were confirmed by unsupervised hierarchal clustering and Pearson’s correlation coefficient analyses (Fig. 1C) and these may be associated with the major EGA of buffalo embryos at the 16-cell stage. The Pearson’s correlation coefficients between the two replicate samples at the same developmental stage were all over 0.91 (Table S4), and this demonstrated there was the high biologically reproducibility between them.

Gene expression differences throughout the buffalo PED were analyzed. From 129 to 6,261 DEGs were detected by pairwise comparisons of any two consecutive developmental stages (Fig. 1D). The trend of DEGs displayed that the number of DEGs was markedly increased at the 8- and 16-cell stages (2,049 genes were up-regulated and 2,249 genes were down-regulated) and the enriched GO terms of these DEGs were involved in those biological processes about translation and ribosome (Fig. S2). The dynamic expression heatmap of DEGs showed that the notable inflection points in the two trend groups were both present at the 16-cell stage (Fig. S3).

The MII oocyte’s gene expression pattern represented the maternal gene expression profile. The expression suppression of maternal genes was investigated (Fig. 1E). A few maternal gene expressions were suppressed from the zygote to the 8-cell stage. However, the number of expression suppression of maternal genes was markedly increased from the 16-cell (69 genes) to the ICM (blastocysts) stage (979 genes). The first expressed genes at each embryonic developmental stage were also identified and calculated using the genes expressed at the GV and MII oocyte stages as baseline (Fig. 1F). After fertilization, the first peak of the first expressed gene number appeared at the 4-cell stage (151 genes), but the most significant peak appeared at the 16-cell stage when 352 genes were first expressed. As critical transcriptional regulators in the oocyte-to-embryo transition [29, 30], the distinct onset of *NANOG* and *SOX2* gene expression was at the 16-cell stage, with very limited transcription occurring at the 4- and 8-cell stages (Fig. S4). These results provided evidence in further analysis of buffalo EGA.

### Construction of the DNA methylome landscape during buffalo PED

In order to further understand the dynamics of DNA methylation during buffalo PED, 9 stages of oocytes or embryos were subjected to trace cell based WGBS. Approximately 12.46 billion raw reads were generated through WGBS. On average, 84.15 GB of clean data were obtained after quality control and the clean reads rate was 93.92%, and the mapping rate was 79.38% (Table S5).

**Fig. 2:**
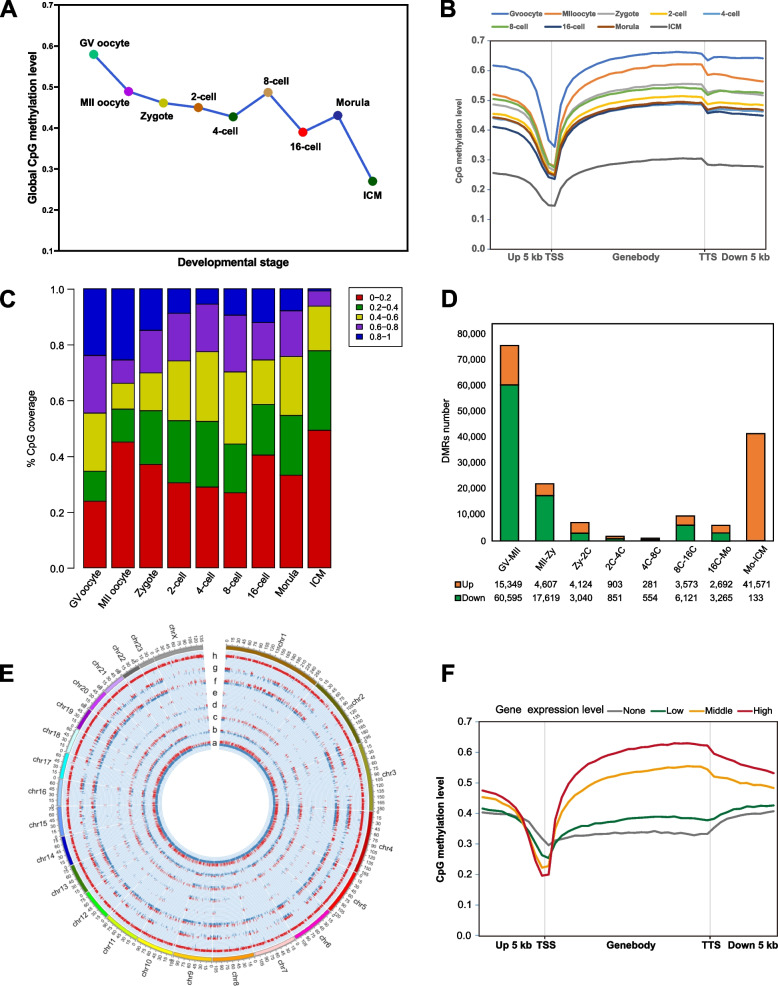
The DNA methylome landscape during buffalo PED. **A** Global CpG methylation levels at each developmental stage. **B** The trend of average CpG methylation levels from 5 kb upstream to 5 kb downstream of the gene bodies. **C** The distribution percentage of CpG with different methylation levels at each developmental stage. **D** The numbers of DMRs in consecutive developmental stages during buffalo PED. **E** The distribution of DMRs across the global genome in several pairwise comparisons. a, GV oocyte vs. MII oocyte; b, MII oocyte vs. Zygote; c, Zygote vs. 2-cell; d, 2-cell vs. 4cell; e, 4-cell vs. 8-cell; f, 8-cell vs. 16-cell; g, 16-cell vs. Morula; h, Morula vs. ICM. **F** The trend of average CpG methylation levels with different expression levels (high, medium, low and no expression) using the 8-cell stage as an example

The global CpG methylation dynamics in buffalo PED were analyzed (Fig. 2A). Following oocyte maturation and fertilization, the global DNA methylation level was significantly decreased from 57.94% in GV oocytes to 42.72% in embryos at the 4-cell stage. The second notable decrease occurred from 48.67% in embryos at the 8-cell stage to 38.97% in embryos at the 16-cell stage, and this suggested that 16-cell stage was correlated with buffalo major EGA. The results were consistent with the decrease in expression of the maintenance of *DNMT1* and the increases in expression of *TET1* and *TET2* from the zygote to the 16-cell stage (Fig. S5). The third sharp decrease occurred from the morula to the ICM, which might be correlated with the differentiation of the ICM and the trophectoderm. Interestingly, we could also observe two global CpG methylation level rises. One occurred at the 8-cell stage and the other was at the morula stage. This finding indicated that global demethylation occurred throughout the whole buffalo PED process, and that de novo methylation was ongoing after EGA.

Subsequently, the DNA methylation levels of 5 kb up- to down-stream in the gene body were examined, and the similar DNA methylation trends were found during all the developmental stages (Fig. 2B). In addition, the proportion of CpG sites with hypo-methylation level (0–20%) was 40.41% at the 16-cell stage, which was much higher than at the 2- (30.45%), 4- (28.93%) and 8-cell (26.89%) stages (Fig. 2C, Table S6). This phenomenon indicated that the 16-cell stage was a critical transition point in buffalo embryo MZT.

The results regarding the DMRs were also consistent with the results relating to the DEGs. The number of DMRs found between the 8- and 16-cell stages was 9,694 (Fig. 2D) which was significantly higher than that between 4- and 8-cell stages. GO enrichment showed that the DMRs were enriched in translation, protein phosphorylation, protein binding and so on (Fig. S6). The transcriptional repression of the embryonic genome as a result of DNA methylation could be relieved through DNA demethylation and, subsequently, major events in EGA was rapidly initiated. This was also evidenced by the genome-wide distribution of methylation levels within the DMRs (Fig. 2E).

To gain further insights into how DNA methylation regulated gene expression, the relationship between DNA methylation and gene expression during buffalo PED was investigated. As previously reported, the DNA methylation levels at the promoter regions were negatively correlated with the expression levels of the corresponding genes (Fig. S7A). Thus, the higher expression levels of genes corresponded to the low methylation levels in the promoter regions and the high methylation levels in the gene body regions (Fig. 2F). The expression of the embryonic genome was negatively correlated with the DNA methylation levels, especially with respect to the promoters, and its activation was regulated by the DNA methylation levels [31].

### Identification of stage-specific co-expression modules by WGCNA

**Fig. 3:**
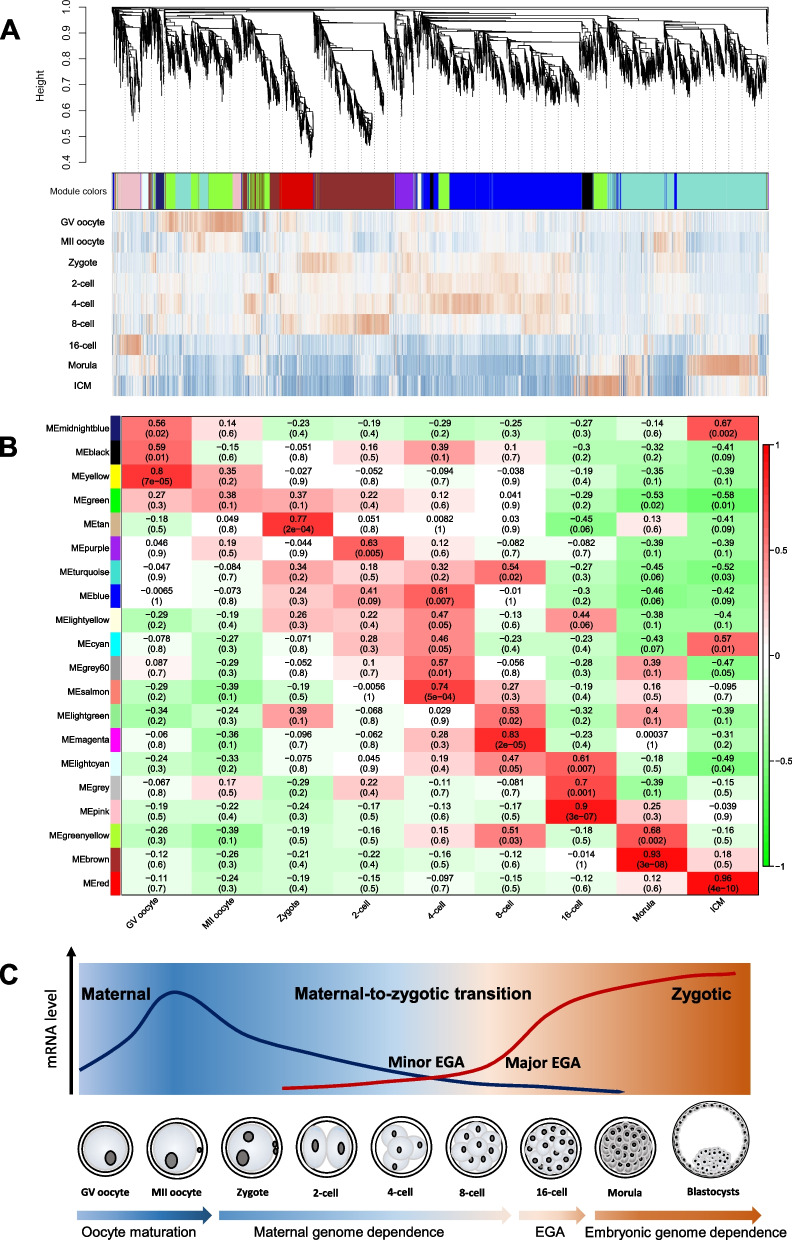
Gene co-expression analysis of stage-specific dynamics by WGCNA. **A** A cluster dendrogram showing the modules of the co-express genes identified. **B** A heatmap of the correlations between the co-express modules and the embryonic stage of development. **C** Schematic diagram of developmental steps during buffalo PED

WGCNA was performed in order to reveal the dynamics of gene co-expression patterns and regulation mechanisms at the whole genome level during buffalo PED. A total of 20 modules of co-expressed modules were identified and a cluster dendrogram for modules was constructed (Fig. 3A). The three modules, pink, lightcyan and grey, had a significant association with the 16-cell stage (Pearson’s correlation coefficient (*r*) > 0.6, *P* < 0.01) (Fig. 3B). It was noted that some stage-specific modules showed significant continuity and were involved in the buffalo MZT process from the 2- to the 8-cell stages, such as turquoise, blue, lightyellow, salmon and magenta modules (Fig. 3B). These results indicated that a strict and continuous program before the 16-cell stage was necessary for buffalo EGA and the step-by-step timing activation of the molecular functions provided a cascade for the events involved in the buffalo embryonic development.

The co-expression pattern of the whole genome (Fig. 3A) was further confirmed the results in transcriptome and DNA methylome analyses. Whole-genome transcriptome and DNA methylome analyses uncovered a series of sequential ordered developmental progression processes during the early embryogenesis of buffaloes. The whole development stages were divided into four steps: oocyte maturation (from GV to MII oocytes), maternal genome dependence (from zygote to 8-cell), EGA (16-cell) and embryonic genome dependence (from morula to blastocyst) (Fig. 3C). In the first step, a large number of maternal mRNA transcription and protein translations were completed and these were stored in the MII oocytes. After fertilization, embryonic development relied on maternal mRNAs and proteins, and these were then gradually degraded [32]. The gene transcription and translation of the buffaloes’ major embryonic genome occurred simultaneously at the 16-cell stage which was the EGA step. This was the most crucial transition period of transcriptional regulation during the early embryogenesis of buffaloes. In the fourth step, embryonic compaction and cell differentiation depended on the embryonic genome.

### Genetic program dynamics during buffalo EGA

**Fig. 4:**
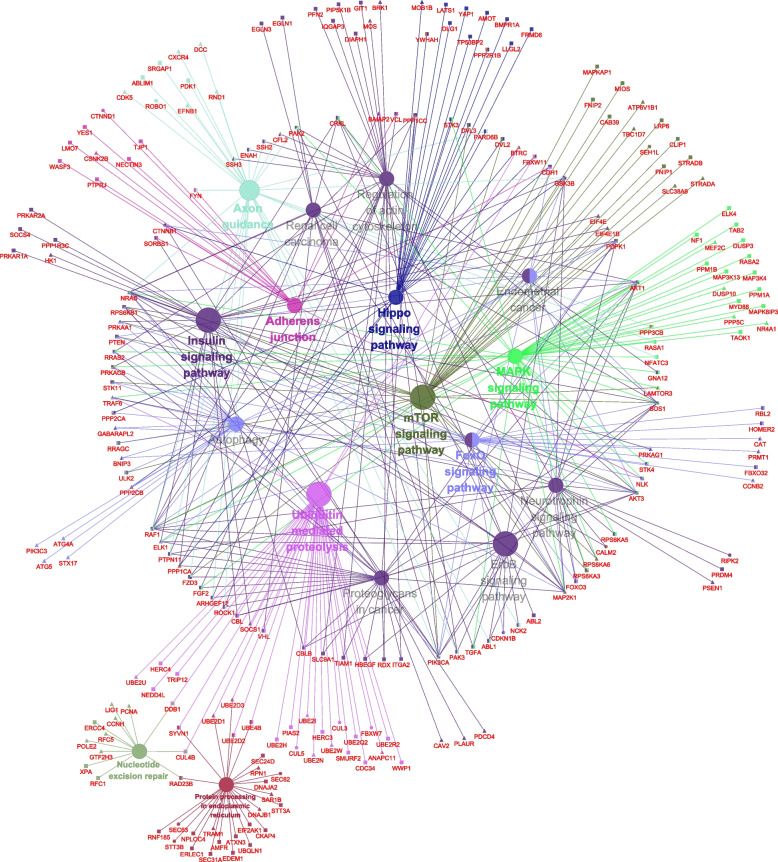
The interaction network of enriched KEGG pathways before major EGA. Hexagon: 2-cell stage; Rectangle: 4-cell stage; Triangle: 8-cell stage

**Fig. 5:**
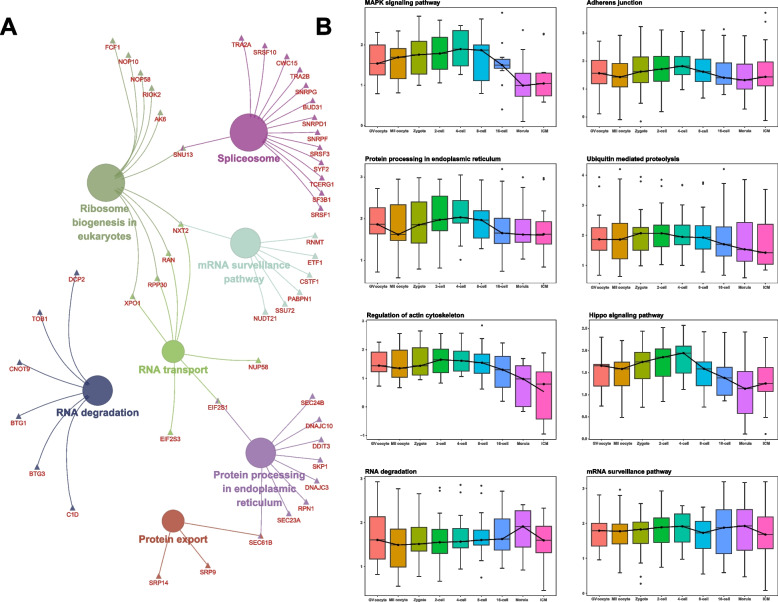
Enriched pathway at the 16-cell stage and the dynamics of the important KEGG pathways. **A** KEGG pathway enrichment at the 16-cell stage. **B** The timing genome-wide activation of key pathways during buffalo PED

To further reveal the sequential developmental progression of the genetic regulatory network in buffalo EGA, functional enrichment analysis of the hub genes in the stage-specific modules were performed. Before major EGA, the buffalo embryos at 2- to 8-cell stages were highly enriched to the nucleotide excision repair pathway, MAPK signaling pathway, mTOR signaling pathway, Hippo signaling pathway, insulin signaling pathways, adherens junction pathway and ubiquitin mediated proteolysis as well as other processes (Fig. 4, Table S7). The enriched signaling pathway at the 16-cell stage mainly included protein processing in endoplasmic reticulum, protein export, spliceosome, RNA transport and RNA degradation (Fig. 5A, Table S7). The timing of genome-wide activation of these signaling pathways (Fig. 5B, S8) indicated that various critical signaling pathways associated with MZT were activated successively after fertilization, and the biological processes associated with EGA such as cell cycle transition, maternal mRNAs degradation, ubiquitin-mediated proteolysis and translation initiation occurred gradually in a specific time sequence (Fig. S9). These results implicated that a series of sequentially ordered waves were essential for buffalo EGA to proceed at the 16-cell stage.

During buffalo MZT, several critical cellular and molecular events were evident. During cell cycle transition, the mitotic cell cycle process showed a gentle upslope from the 2- to the 8-cell stages and this was accompanied by the activation of protein serine/threonine kinase activities, and the changes caused the transition of the cell cycle G2/M phase (Fig. S8, S9A). As one of the key mechanisms underlying cell cycle control [33], ubiquitin-mediated proteolysis and ubiquitin-protein transferase and ligase exhibited a similar trend (Fig. 5, S9C). At the same time, the cAMP signaling pathway showed a largely opposite trend (Fig. S8). During the translation event, which is another critical cellular and molecular event, the 16-cell stage was the significant turning point. At this time, the translation factor activity, translation initiation factor activity and translational initiation all showed downward trends from the 2- to the 8-cell stages and an upward trend after the 16-cell stage. However, the opposite trend was observed for the regulation of gene silencing (Fig. S9D). These results corroborated with previous studies that the early embryonic developmental capacity before EGA was mainly governed by maternal factors, including several mRNAs and proteins. In addition, it was noted that gene *CNOT7* was enriched in the biological process of the CCR4-NOT complex and this was considered to be a key regulator for maternal mRNA degradation [34], and it was highly expressed at the 2- to 8-cell stages (Fig. S9E).

### Identification of the key hub genes

**Fig. 6:**
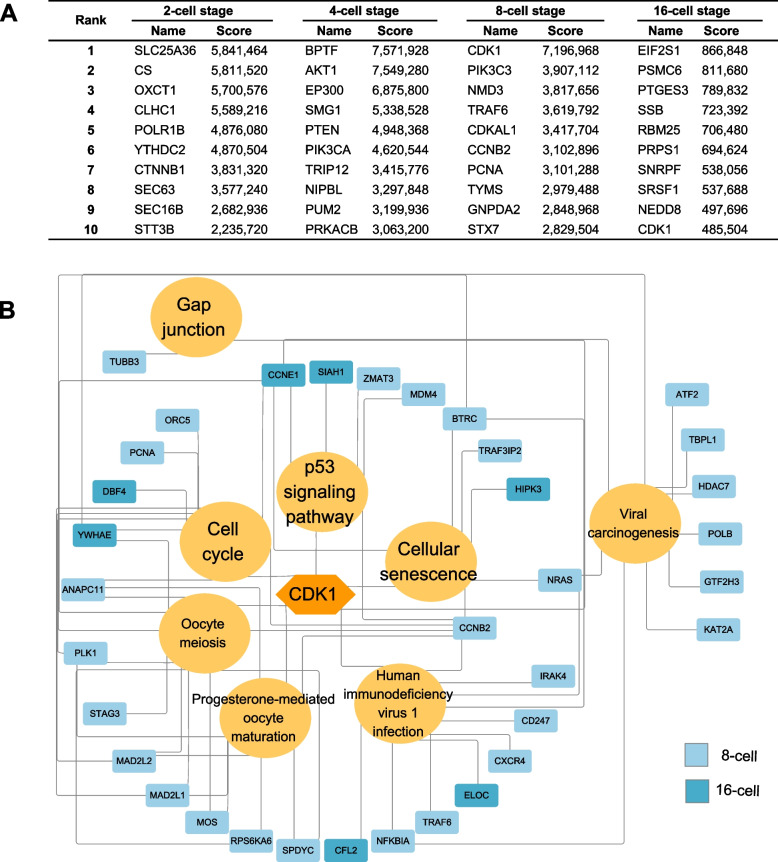
Identification of the key hub genes during MZT. **A** The list of top 10 hub genes. **B** regulation pathways and related hub genes of CDK1 during EGA

**Fig. 7:**
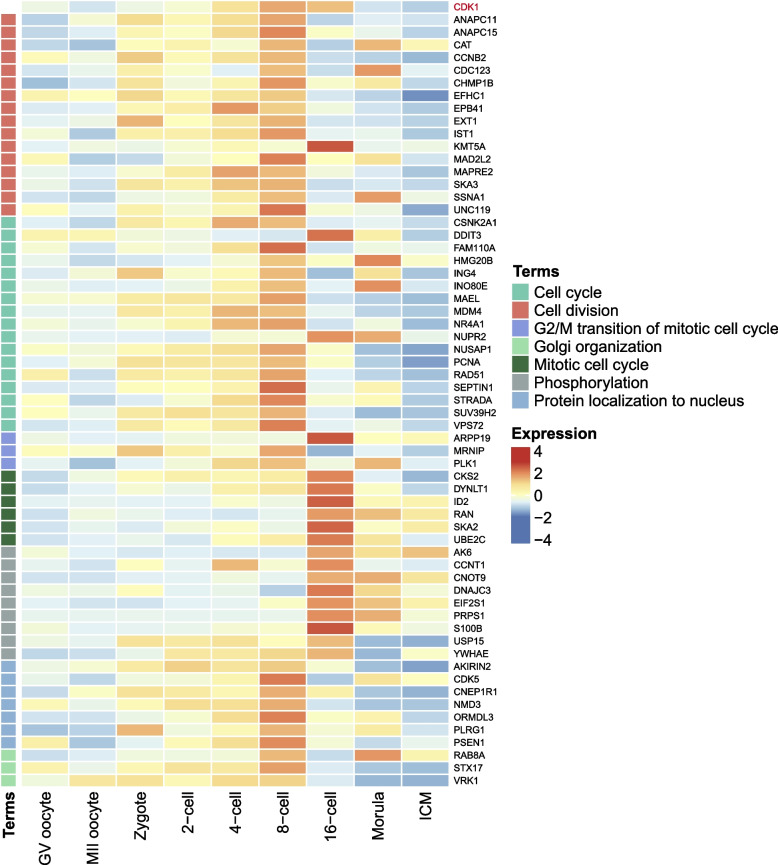
Expression heatmap of enriched biological processes of CDK1

According to cytoHubba analysis for the top 10 hub genes at each buffalo embryonic developmental stage, some essential genes were mined that appeared to play critical roles in the regulation of the cell cycle, cell survival and proliferation, translation, mitosis and mRNA decay. These included *CDK1*, *AKT1*, *EIF2S1*, *CDH1* and *SMG1*. Notably, *CDK1* was identified as one of the top 10 hub gene at both the 8- and 16-cell stages (Fig. 6A). *CDK1* participated in the regulation of buffalo EGA mainly by cell cycle, gap junction, cellular senescence and p53 signaling pathway (Fig. 6B). After fertilization, the expression of *CDK1* and the related hub genes began to be upregulated expression to prepare for buffalo EGA by regulating cell cycle. The cell cycle was observed to completed the G2/M transition at 8- to 16-cell stages (Fig. 7). *CDK1* also was involved in protein localization to nucleus before major EGA, but it also took part in phosphorylation modification at the 16-cell stage. In order to validate the repeatability and reproducibility of the sequencing data, the qRT-PCR experiments were performed. The qRT-PCR results of *CDK1* measurements were in agreement with the RNA-seq sequencing results (Fig. S10A). In addition, *CNOT7*, *BMP15*, *GDF9* and *HDAC7* were also enriched in some important biological processes during buffalo EGA (Table S8).

## Discussion

In mammals, PED is a highly dynamic and delicately orchestrated process that involves various biological events in which gene expression is dramatic, systematic, orchestrated and regular [35]. Along with the gradual degradation of maternal transcripts by maternal (M)-decay and zygotic (Z)-decay pathways [36], the embryonic genome is activated and begins to be responsible for the regulation of embryonic development during MZT. Inhibition of zygotic expression of *TUT4/7* impaired Z-decay process, and leaded to early embryonic developmental arrest [4]. Embryos are sensitive to the environment during MZT and this is related to successful EGA, and easy to arrest at this stage in vitro. Therefore, an in-depth exploration of the buffalo EGA and genetic programming during buffalo PED will be valuable to understand the molecular mechanism of buffalo embryogenesis and this will lay a foundation for improving the in vitro development of buffalo embryos.

Early research on embryonic EGA mainly adopted the way using α-amanitin to treat zygotes to inhibit transcription, but the experiment confirmed that not all embryonic transcription were completely inhibited for different genetic backgrounds [37]. The reason why buffalo embryonic development was blocked at the 8-cell stage [10] might be due to the inhibition of minor EGA transcripts during α-amanitin treatment [38]. RNA-seq technology provides a novel strategy for the in-depth deeply understanding of the mechanisms and characteristics of early embryonic development by detecting the whole-genome expression profiling [39]. Transcriptional regulation is largely controlled by DNA methylation. A drastic DNA methylation reprogramming occurs during PED, including global demethylation and remethylation [40]. Therefore, in our study, we performed RNA-seq and WGBS across 9 developmental stages in order to characterize the global transcriptional profile and DNA methylation landscape of buffalo pre-implantation embryonic, thus revealing the in-depth mechanisms of buffalo EGA and the regulatory network of MZT.

Our study comprehensively showed the dynamics of transcriptional profiling during buffalo PED. Combined with the confirmation of DNA methylation landscape analysis results, we concluded that the timing of buffalo major EGA occurred at the 16-cell stage. Although in buffalo the major EGA occurred at the 16-cell stage, analysis of the results of the regulation network demonstrated that buffalo MZT was a complex and stepwise process, and the regulatory pathway programs were intertwined to govern gene expression in a systematic and ordered manner [41]. Some pathways were activated during the 2- to 8-cell stages and these acted as the pioneer signals in preparation for the EGA process [42], and then a larger number of embryonic genes were activated at the 16-cell stage. This preparation process was found to be longer than those seen in many other animals, and this might be one of the reasons for the low fecundity observed in buffaloes.

In our study, many important signaling pathways were enriched during the buffalo MZT. For example, the Hippo signaling pathway could control Z-decay activation of maternal mRNAs [36] by being involved in the selective mRNA 3’-oligouridylation of short-tailed maternal mRNAs [43] and then these could activate embryonic genome. Moreover, the Hippo signaling pathway could also be interconnected with key signaling cascades of several other signaling pathways which can affect embryo development, such as the Wnt, Notch and TGF-β signaling pathways. Notably, cross-talk among multiple pathways (including the MAPK, mTOR, FoxO and insulin signaling pathways and autophagy) were relatively quite significant before the buffalo major EGA stages. *AKT1* and *AKT3* were the core genes in the cross-talk of these pathways and these encode the serine/threonine kinases. Upregulation of *PTEN* could also induce autophagy by inhibiting the PI3K-AKT-mTOR pathway [44]. In buffalo embryos, the mTOR signaling and autophagy pathways also shared multiple genes, such as *MAP2K1*, *PDPK1*, *PIK3CA*, *RAF1*, *RPS6KB1*, *RRAGC*, *STK11* and *ULK2*. These interaction mechanisms require further exploration in the future.

As the hub genes in buffaloes MZT, *CDK1* played vital roles in various biological processes. *CDK1* and cyclin B1 formed the major mitotic kinases that are involved in cell cycle-dependent binding during mitosis [45]. *CDK1* activity was regulated by the cooperativity of the inhibitory kinase *Wee1* and the activating phosphatase *CDC25*, and may be involved in mitotic entry by inhibiting and stabilizing the binding of microtubules to the kinetochores [46]. Cells were shown to be arrested at the G2 phase in the presence of *CDK1* inhibitors [47]. *CDK1* was also involved in cell cycle arrest in the G2/M phase before EGA by p53 pathway [48]. One of the reasons of why the timing of buffalo major EGA was later than that of other animals would be that G2/M transition of mitotic cell cycle was delayed to 16-cell stage. As the other ‘Master Regulator’ of autophagy [49], *CDK1* could regulated mitotic cell cycle progression by inducing the phosphorylation of some autophagy-related proteins such as *ULK1* and *ATG13* [50]. The resultant cellular senescence might be a potential key factor that affect buffalo embryo MZT. *CDK1* could improve global protein synthesis in proliferating cells by activating 5’ terminal oligopyrimidine mRNA translation. It was also able to form a homeostatic network with mTOR and Ras/Erk in order to coordinate cell proliferation and protein synthesis [51]. Therefore, *CDK1* could regulate buffalo EGA through multiple pathways.

## Conclusions

In this novel study we sequenced the global transcriptomes and DNA methylation in buffalo oocytes and pre-implantation embryos by using RNA-seq and WGBS techniques, and this allowed us to draw the whole-genome transcription and DNA methylation landscapes for these processes in buffalo embryogenesis. Based on the dynamic characteristics of genes expression and DNA methylation changes, buffalo major EGA was confirmed to occur at the 16-cell stage of embryo development. During buffalo MZT, the sequential activation of key genes and signaling pathways were essential for buffalo EGA and genetic programming of buffalo PED. This study will provide important additional information for understanding molecular mechanisms of buffalo embryogenesis and lay a foundation for improving the in vitro development of buffalo embryos.

## Supplementary Information

**Additional file 1: Fig. S1.** The number of co-expressed genes and exclusively expressed genes at each developmental stage.

**Additional file 2: Fig. S2.** GO enrichment of the DEGs between 8- and 16-cell stages. **A** Up-regulated DEGs. **B** Down-regulated DEGs.

**Additional file 3: Fig. S3.** Expression heatmap of DEGs during buffalo PED.

**Additional file 4: Fig. S4.** The Sashimi plot of NANOG (**A**) and SOX2 (**B**) at respective developmental stages.

**Additional file 5: Fig. S5.** Expression heatmap of the genes related DNA methylation and demethylation.

**Additional file 6: Fig. S6.** GO enrichment of DMRs between 8- and 16-cell stages. **A** Up-regulated DMRs. **B** Down-regulated DMRs.

**Additional file 7: Fig. S7.** Coefficients of Pearson correlation (*r*) between DNA methylation levels in different regions (red curves) and relative expression levels of respective genes (yellow curves). **A** In promoter regions. **B** In gene body regions.

**Additional file 8: Fig. S8.** The dynamic patterns of the important KEGG pathways at different developmental stages.

**Additional file 9: Fig. S9.** The timing genome-wide activation of the molecular biological processes during buffalo PED. **A** GO terms related with the cell cycle. **B** GO terms related with protein modification and stability. **C** GO terms related with ubiquitin protease. **D** GO terms related with translation. **E** GO terms related with mRNA catabolism.

**Additional file 10: Fig. S10.** Validation of the gene expression profile of RNA-seq data by qRT-PCR.

**Additional file 11: Table S1.** Sample numbers and collection times for each sample used in this study.

**Additional file 12: Table S2.** The primer sequences for qRT-PCR.

**Additional file 13: Table S3.** QC and mapping summary of RNA-seq data.

**Additional file 14: Table S4.** The matrix of Pearson’s correlation coefficients of all samples.

**Additional file 15: Table S5.** QC and mapping summary of WGBS data.

**Additional file 16: Table S6.** The distribution percentage of CpG with different methylation levels at each developmental stage.

**Additional file 17: Table S7.** KEGG enrichment results during MZT.

**Additional file 18: Table S8.** GO BP enrichment results during MZT.

## Data Availability

All the gene expression and DNA methylation data were deposited at NCBI Short Reads Archive with BioProject accession number PRJNA646061.

## Abbreviations

BP: Biological process
COCs: Cumulus-oocyte complexes
DEGs: Differentially expressed genes
DMRs: Differentially methylated regions
EGA: Embryonic genome activation
GO: Gene Ontology
GV oocyte: Germinal Vesicle oocyte
ICM: Inner cell mass
IVC: In vitro culture
IVEP: In vitro embryo production
IVF: In vitro fertilization
IVM: In vitro maturation
KEGG: Kyoto Encyclopedia of Genes and Genomes
MII oocyte: Metaphase II oocyte
MZT: Maternal-to-zygote transition
PCA: Principal component analysis
PED: Pre-implantation embryonic development
SCNT: Somatic cell nuclear transfer
WGBS: Whole-genome bisulfite sequencing
WGCNA: Weighted gene co-expression network analysis
Z-decay: Zygotic decay
