# Supplementary figures and images for: Whole-genome transcriptome and DNA methylation dynamics of pre-implantation embryos reveal progression of embryonic genome activation in buffaloes
Source: J Anim Sci Biotechnol. 2023 Jul 11;14:94. doi: 10.1186/s40104-023-00894-5 (PMC10334608; doi:10.1186/s40104-023-00894-5)

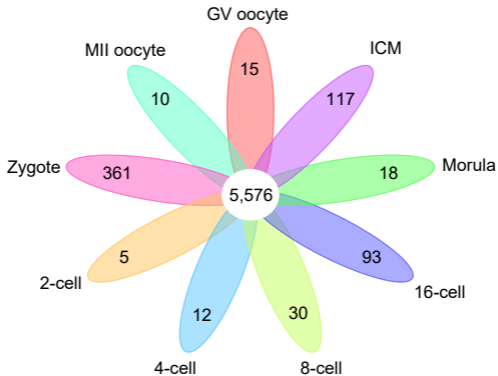

Supplement: Supplementary file 1 — Additional file 1: Fig. S1. The number of co-expressed genes and exclusively expressed genes at each developmental stage. [file 40104_2023_894_MOESM1_ESM.pdf]

Biological process

Molecular function

Cellular component

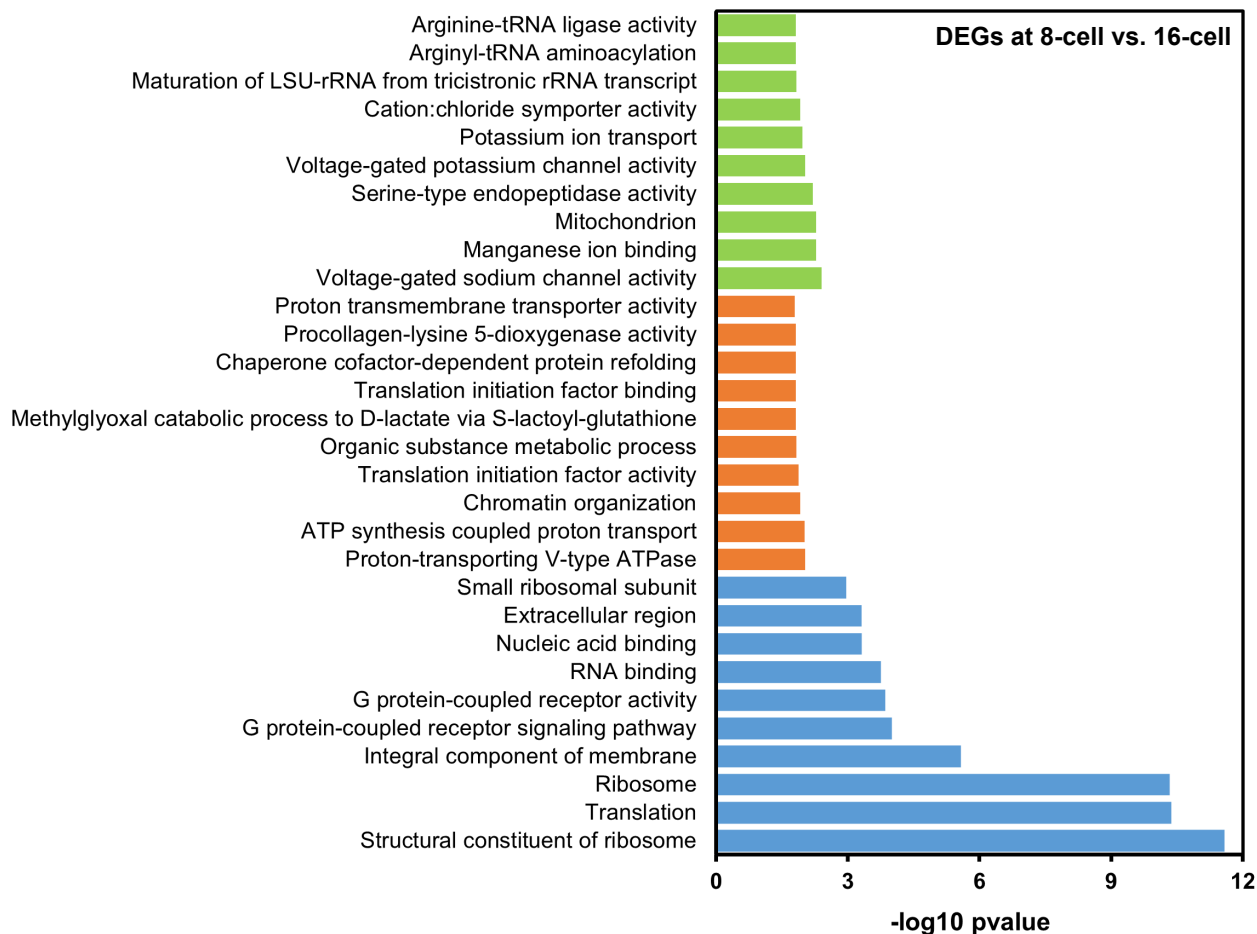

Supplement: Supplementary file 2 — Additional file 2: Fig. S2. GO enrichment of the DEGs between 8- and 16-cell stages. A Up-regulated DEGs. B Down-regulated DEGs. [file 40104_2023_894_MOESM2_ESM.pdf]

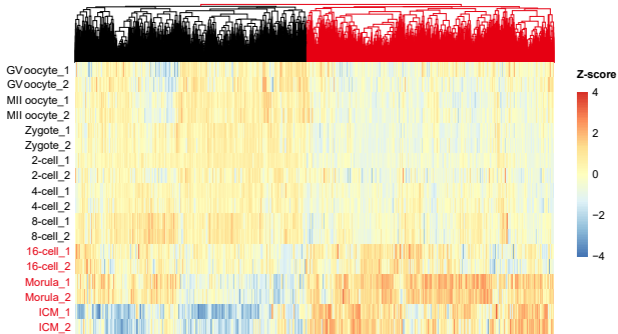

Supplement: Supplementary file 3 — Additional file 3: Fig. S3. Expression heatmap of DEGs during buffalo PED. [file 40104_2023_894_MOESM3_ESM.pdf]

**A**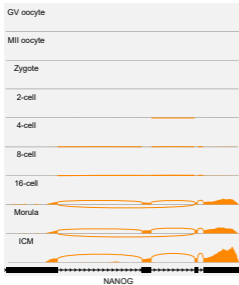**B**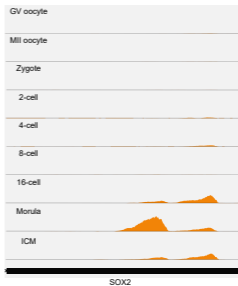

Supplement: Supplementary file 4 — Additional file 4: Fig. S4. The Sashimi plot of NANOG (A) and SOX2 (B) at respective developmental stages. [file 40104_2023_894_MOESM4_ESM.pdf]

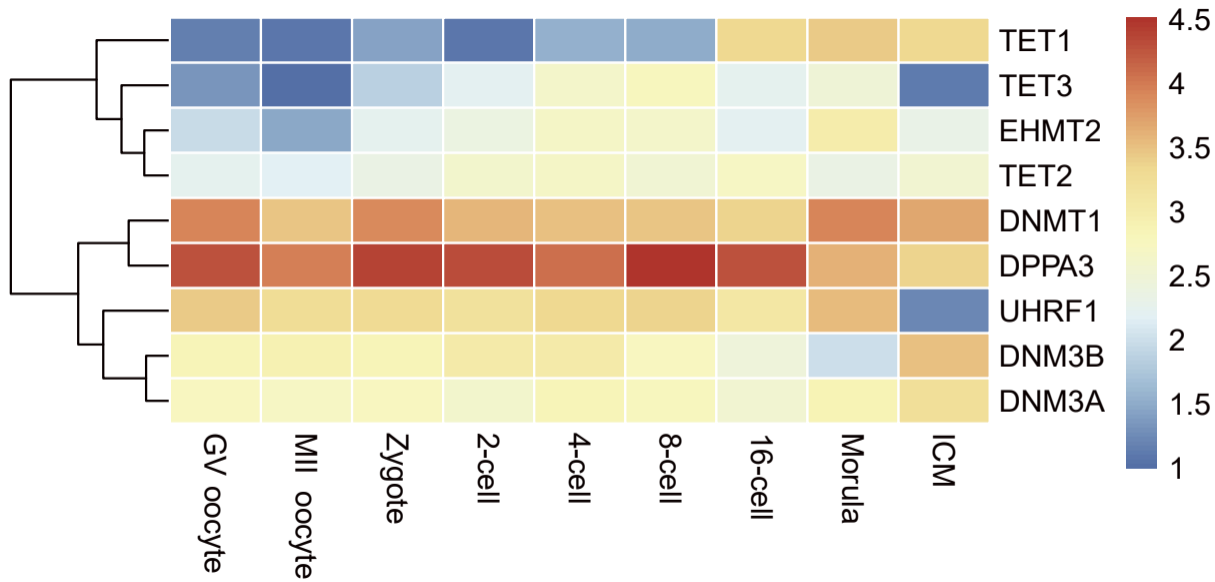

Supplement: Supplementary file 5 — Additional file 5: Fig. S5. Expression heatmap of the genes related DNA methylation and demethylation. [file 40104_2023_894_MOESM5_ESM.pdf]

■ Biological process

■ Molecular function

■ Cellular component

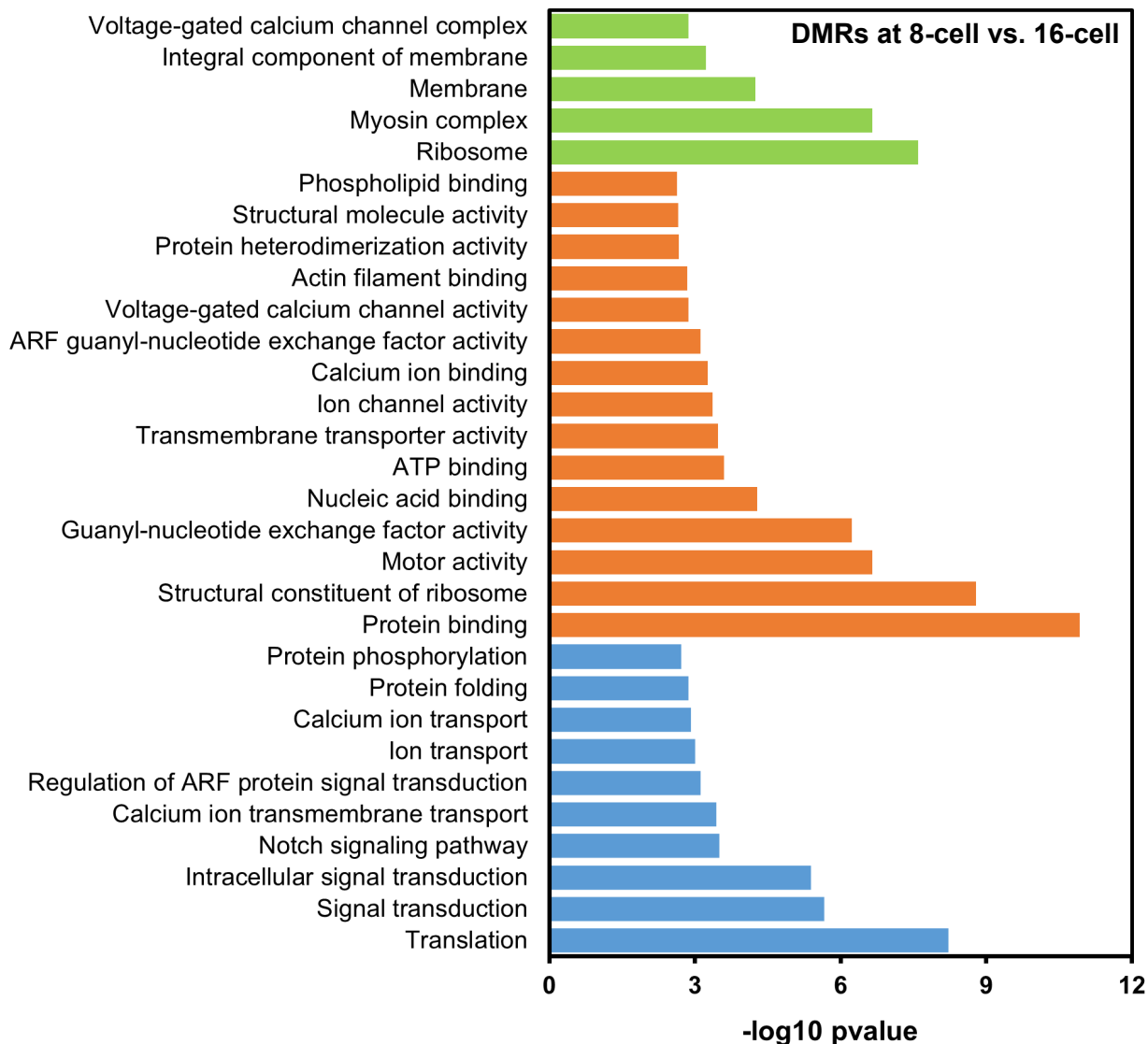

Supplement: Supplementary file 6 — Additional file 6: Fig. S6. GO enrichment of DMRs between 8- and 16-cell stages. A Up-regulated DMRs. B Down-regulated DMRs. [file 40104_2023_894_MOESM6_ESM.pdf]

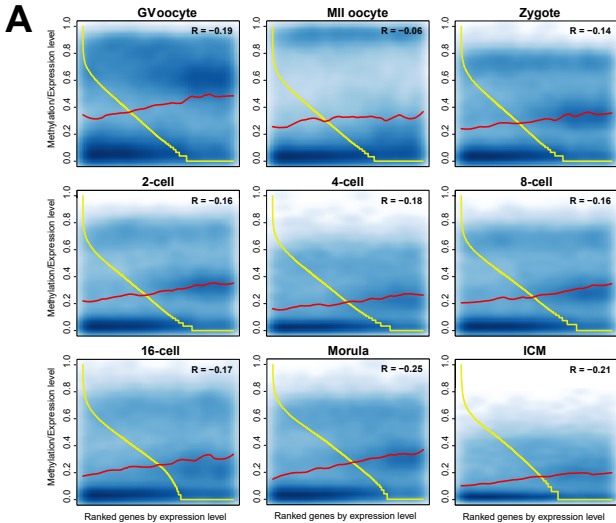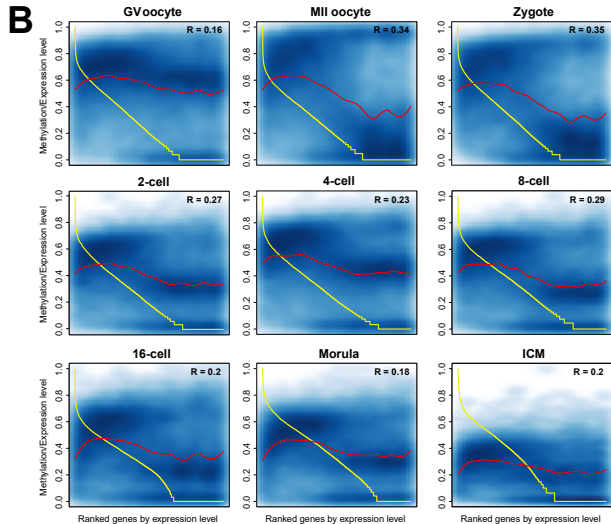

Supplement: Supplementary file 7 — Additional file 7: Fig. S7. Coefficients of Pearson correlation (r) between DNA methylation levels in different regions (red curves) and relative expression levels of respective genes (yellow curves). A In promoter regions. B In gene body regions. [file 40104_2023_894_MOESM7_ESM.pdf]

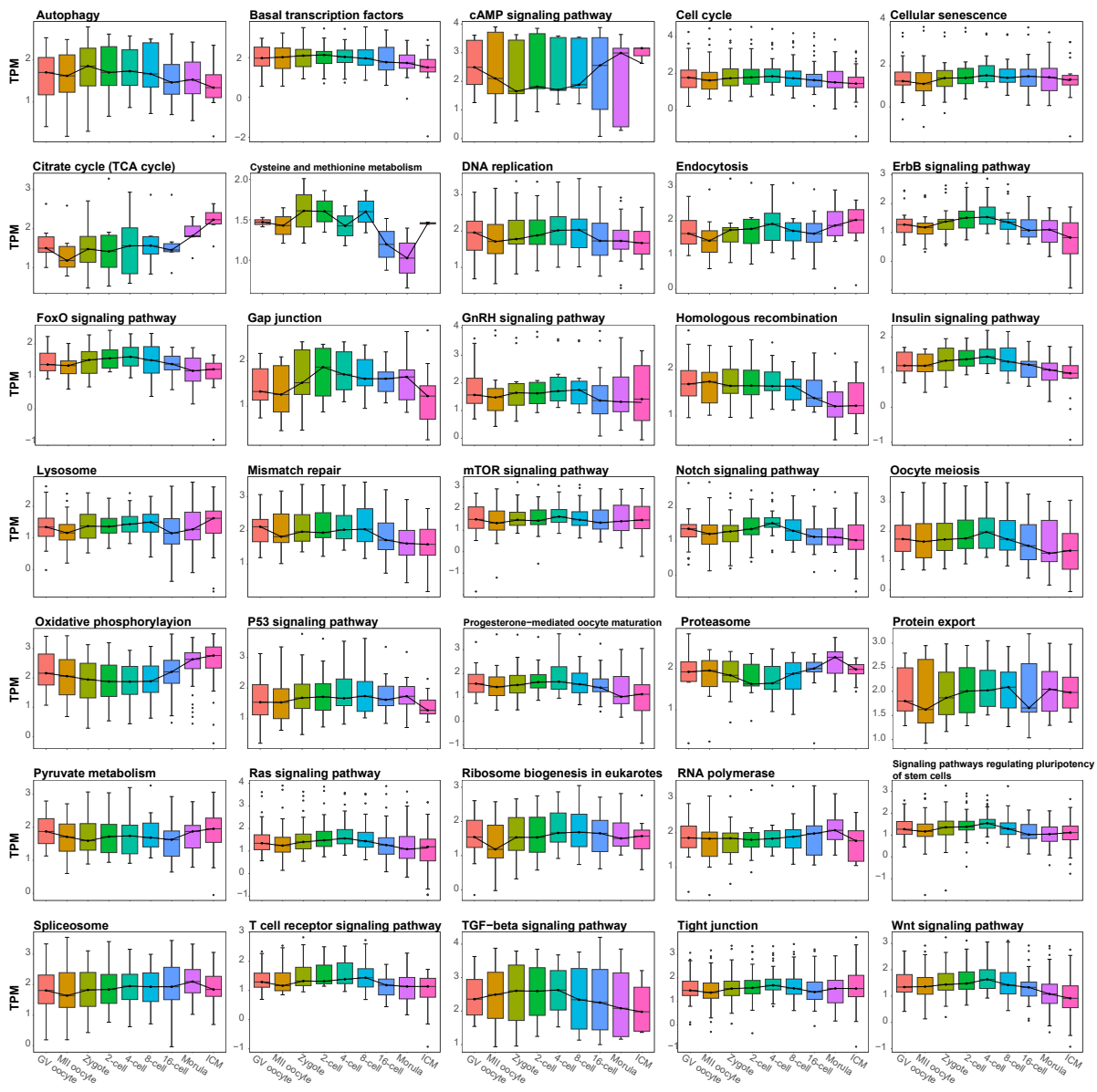

Supplement: Supplementary file 8 — Additional file 8: Fig. S8. The dynamic patterns of the important KEGG pathways at different developmental stages. [file 40104_2023_894_MOESM8_ESM.pdf]

**A**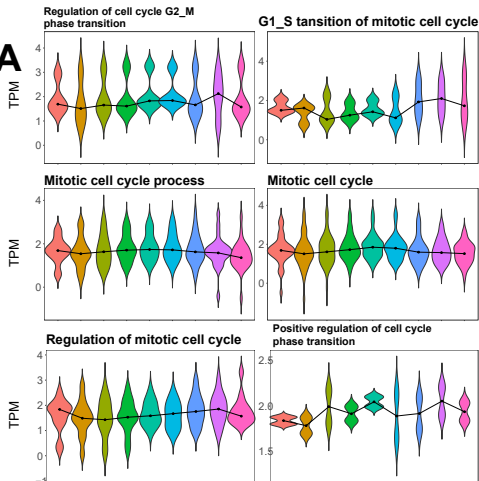**B**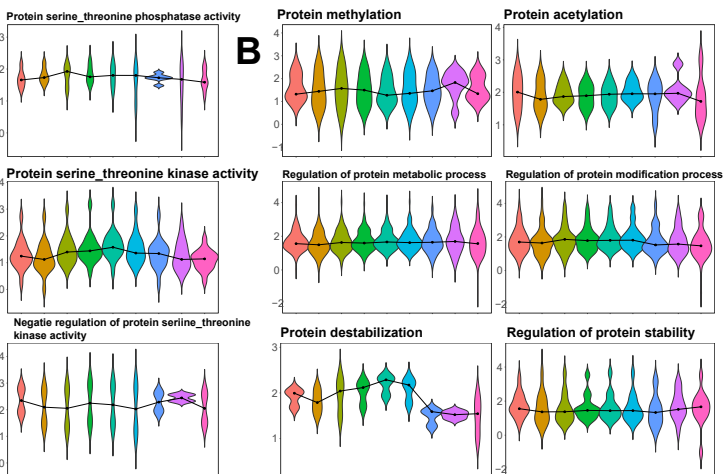**C**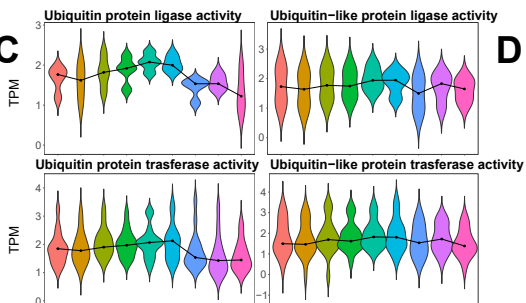**D**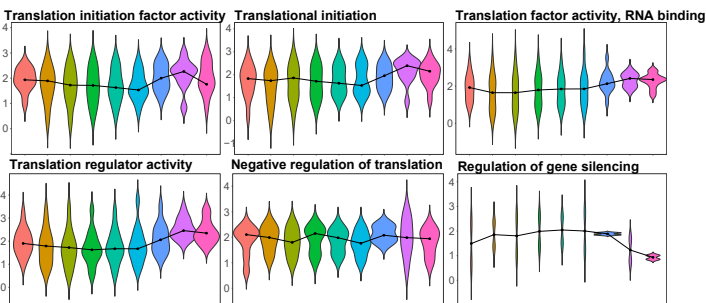**E**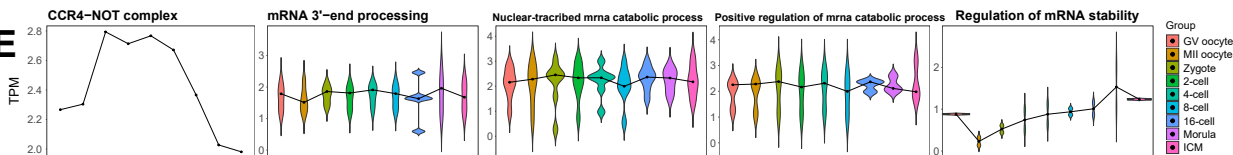

Supplement: Supplementary file 9 — Additional file 9: Fig. S9. The timing genome-wide activation of the molecular biological processes during buffalo PED. A GO terms related with the cell cycle. B GO terms related with protein modification and stability. C GO terms related with ubiquitin protease. D GO terms related with translation. E GO terms related with mRNA catabolism. [file 40104_2023_894_MOESM9_ESM.pdf]

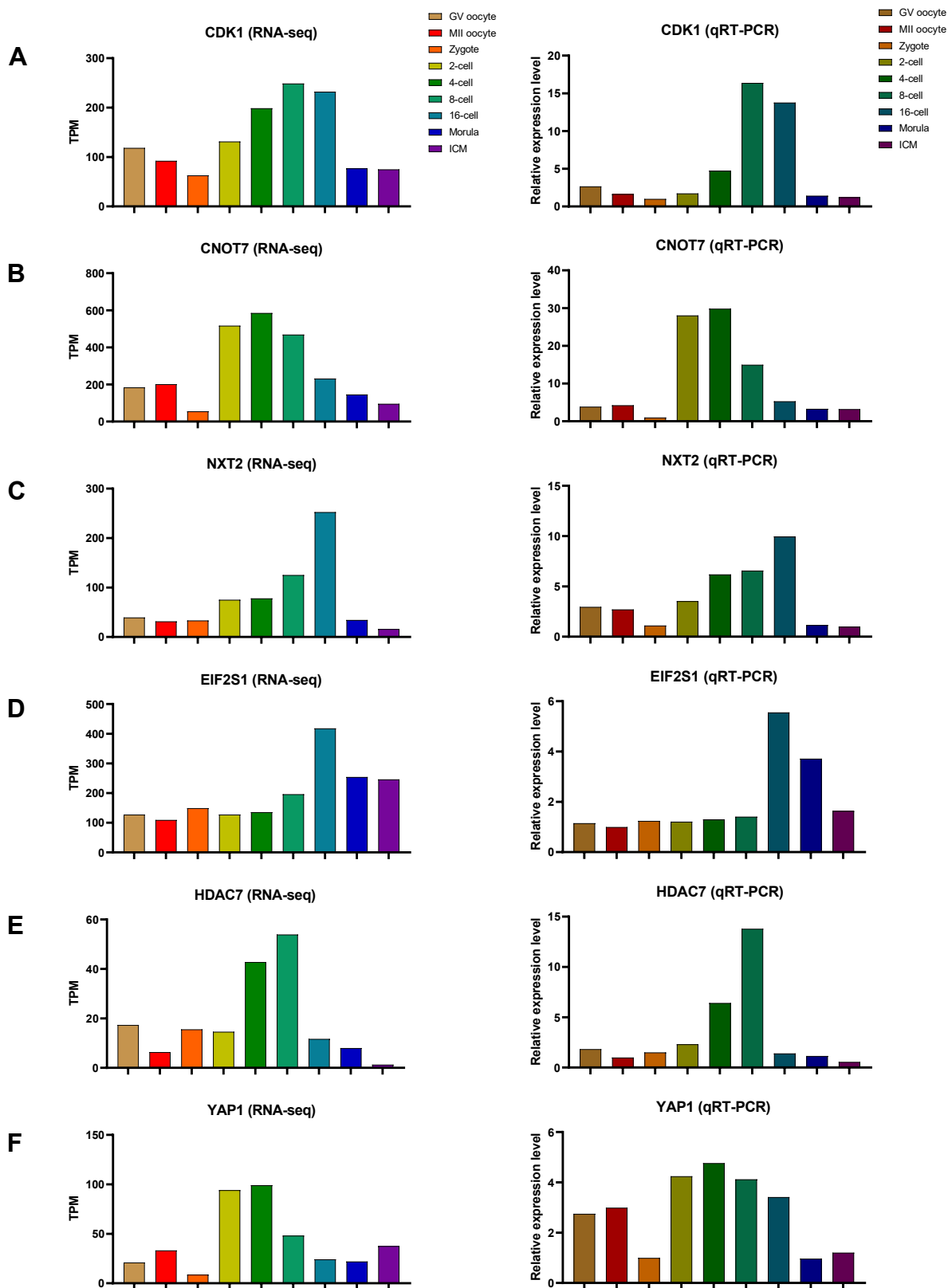

Supplement: Supplementary file 10 — Additional file 10: Fig. S10. Validation of the gene expression profile of RNA-seq data by qRT-PCR. [file 40104_2023_894_MOESM10_ESM.pdf]
